# Incidence and risk factors for occult lesions in low-risk papillary thyroid microcarcinoma patients with tumor characteristics appropriate for thermal ablation: A retrospective study

**DOI:** 10.1097/MD.0000000000034938

**Published:** 2023-09-22

**Authors:** Langping Jin, Kaijun Zhu, Changliang Xu, Jiaying Lu, Liming Huang

**Affiliations:** a Department of Breast and Thyroid Surgery, Shaoxing People’s Hospital, Shaoxing, China; b Department of Ultrasound, Shaoxing People’s Hospital, Shaoxing, China.

**Keywords:** central lymph node metastasis, contralateral occult carcinoma, ipsilateral occult carcinoma, papillary thyroid microcarcinoma, risk factors, thermal ablation

## Abstract

In recent years, thermal ablation has been increasingly employed for the treatment of low-risk papillary thyroid microcarcinoma (PTMC) across various institutions. Its use as a standard or initial treatment continues to be a subject of debate. Retrospective analyses of the surgical pathology in post-ablation patients have indicated that occult lesions are not uncommon. This retrospective study aimed to examine the incidence and risk factors of occult lesions via postoperative pathology in low-risk PTMC patients who fulfilled the criteria for thermal ablation therapy. We examined the medical records of patients who underwent thyroid surgery and had a Bethesda classification V or VI based on fine needle aspiration cytology between November 22, 2020, and December 31, 2022. A total of 413 patients with preoperative tumor characteristics appropriate for thermal ablation were included in this study. Occult lesions, encompassing ipsilateral or contralateral occult carcinoma or central lymph node metastases may have occurred in 34.7% of patients. Male gender (OR: 2.526, 95% CI: 1.521–4.195, *P* = .000), tumor location in the lower pole (OR: 1.969, 95% CI: 1.186–3.267, *P* = .009), multiple microcalcifications (OR: 5.620, 95% CI: 2.837–11.134, *P* = .000), and Hashimoto’s thyroiditis (OR: 2.245, 95% CI: 1.292–3.899, *P* = .004) were independent risk factors for the presence of occult lesions. In low-risk PTMC patients exhibiting tumor characteristics amenable to thermal ablation, over one-third of the patients may present with occult lesions. Meticulous evaluation of the presence of additional lesions is necessary before performing thermal ablation, particularly in patients exhibiting high-risk factors for occult lesions.

## 1. Introduction

In recent decades, the prevalence of papillary thyroid carcinoma (PTC) has experienced a significant surge, with papillary thyroid microcarcinoma (PTMC), characterized by a diameter ≤ 10 mm, being the most rapidly increasing subtype within the PTC population.^[1]^ The majority of PTMC cases exhibit slow progression and favorable prognoses. An investigation utilizing the National Cancer Institute’s Surveillance Epidemiology and End Results database revealed that 18,445 patients with surgically confirmed PTMC demonstrated an exceptional overall prognosis, boasting 10-year and 15-year disease-specific survival rates of 99.5% and 99.3%, respectively.^[2]^ Nevertheless, it has been reported that 19% of PTMC cases may present advanced characteristics, such as central lymph node metastasis (CLNM), lateral lymph node metastasis, gross extrathyroidal extension, and distant metastasis, all of which are associated with significantly reduced survival rates.^[3]^ Consequently, optimizing the management of PTMC has emerged as a primary focus of discussion among global experts in recent years.

Surgery remains the gold standard of PTMC treatment. Nevertheless, given the slow progression of most PTMC, conventional surgery may not be appropriate for all patients due to potential complications, surgical scarring, lifelong levothyroxine replacement post-operatively, and negative impacts on the quality of life.^[4]^ In recent years, alternative approaches, such as thermal ablation, have been increasingly employed for non-surgical PTMC treatment across various institutions, including radiofrequency ablation, microwave ablation, and laser ablation. Thermal ablation is a minimally invasive PTMC treatment that offers safety, expedited postoperative recovery, enhanced economic benefits, and a superior postoperative quality of life.^[5–9]^ A recent meta-analysis comprising 4 retrospective studies comparing thermal ablation and surgical outcomes in low-risk PTMC patients revealed no local recurrence or distant metastasis in either group during an average follow-up period of 3 years.^[10]^ Consequently, thermal ablation may serve as a viable alternative for low-risk PTMC patients who are either unwilling or unable to undergo surgery.^[11–14]^ Nonetheless, PTMC is susceptible to occult lesions, including ipsilateral or contralateral occult carcinoma and occult lymph node metastases, and radical tumor eradication may not be achieved through thermal ablation in such patients. Retrospective analyses of surgical pathology in post-ablation patients have indicated that occult lesions are not uncommon.^[15–18]^ These occult lesions may become sources of future disease recurrence or even distant metastases. As a result, thermal ablation remains a non-radical treatment for low-risk patients with PTMC, and its utilization as a standard or initial treatment continues to be a subject of debate.

In an effort to enhance the diagnosis and management of PTMC while standardizing the therapeutic approach for thermal ablation, the Ultrasound Doctor Branch of the Chinese Medical Doctor Association has established the “Expert Consensus on Indications for Thermal Ablation in the Treatment of Papillary Thyroid Microcarcinoma.”^[19]^ The criteria included non-pathological high-risk subtypes, ultrasound indicative of a solitary suspicious nodule with a maximum diameter ≤ 1 cm, absence of coarse calcifications within the nodule, tumor not infiltrating the thyroid capsule, no evidence of lymph node or distant metastases, among others. Given the slow progression of most low-risk PTMC patients and the median time to recurrence is 10 years,^[20]^ the follow-up duration for existing thermal ablation patients remains brief, and long-term efficacy and recurrence rates are yet to be determined. The incidence and risk factors for occult lesions in low-risk PTMC patients eligible for thermal ablation are uncertain.

Consequently, this study aimed to examine the incidence and risk factors of occult lesions via postoperative pathology in low-risk PTMC patients who fulfilled the criteria for thermal ablation therapy.

## 2. Materials and methods

### 2.1. Patient selection

This retrospective study was approved by the Academic Ethics Committee of Shaoxing People’s Hospital (2021-K-Y-43-01). Because the data are anonymous, written informed consent for participation was not required. We examined the medical records of patients who underwent thyroid surgery and had Bethesda classification V or VI based on fine needle aspiration (FNA) cytology between November 22, 2020, and December 31, 2022. The following criteria were fulfilled for inclusion: preoperative FNA cytology did not indicate aggressive PTC variants (e.g., hobnail, tall cell, columnar, solid) or other thyroid cancer types; preoperative ultrasound assessment of a single suspicious nodule with a maximum diameter of ≤ 1 cm, without coarse calcifications within the nodule, thyroid capsular invasion or extrathyroidal extension (Chinese-Thyroid Imaging Reporting and Data System Category 4A, 4B, 4C, or 5 defined as a suspicious nodule);^[21]^ no evidence of lymph node or distant metastasis on preoperative imaging; no family history of thyroid cancer; no history of radiation exposure to the neck during adolescence or childhood. The exclusion criteria were as follows: any supplementary examination or physical examination suggesting potential lymph node metastasis in the neck or distant metastasis and preoperative imaging evaluation with ≥ 2 suspicious nodules.

### 2.2. Main measurement parameters

Two radiologists with more than 10 years of experience in thyroid ultrasound examinations retrieved patients’ preoperative ultrasound reports and images from the Picture Archiving and Communication System, analyzed them, and documented relevant parameters, including the maximum diameter of the suspicious nodule, location, orientation, margin, distance of the nodule from the capsule, presence of multiple microcalcifications within the nodule, the existence of multiple nodules in the thyroid, and whether the thyroid gland exhibited diffuse enlargement. Multiple microcalcifications were defined as more than 5 punctate hyperechoic foci (diameter, ≤ 1 mm) within the solid component of the nodules. Thyroid nodules adjacent to the capsule were defined as those with a distance ≤ 2 mm from the capsule, but without extending to the thyroid capsule. Preoperative TgAb and TPOAb titers were measured using an electrochemiluminescent immunoassay kit. The normal ranges for TgAb and TPOAb at our institution are 0 to 4.11 IU/mL and 0 to 5.61 IU/mL, respectively. We diagnosed patients with Hashimoto’s thyroiditis (HT) preoperatively if ultrasonography revealed a diffusely enlarged thyroid with abundant blood flow, along with TgAb > 4.11 IU/mL or TPOAb > 5.61 IU/mL.

### 2.3. FNA, DNA isolation, and molecular testing

Ultrasound-guided FNA specimens obtained from patients were separated into consecutive FNA biopsy specimens, and FNA washout precipitates were discarded. Following FNA, the needle and syringe were rinsed with 1 mL normal saline. The discarded FNA washout precipitate was preserved in the ThinPrep PreservCyt® solution. DNA was extracted from FNA washout precipitate samples using a blood DNA extraction kit (Aidlab Biotechnologies Co., Ltd, Beijing, China) according to the manufacturer’s instructions. The concentration and purity of the extracted DNA were determined using a Nanodrop spectrophotometer (Thermo Fisher Scientific, Waltham, MA). BRAF V600E mutations and telomerase reverse transcriptase (TERT) promoter mutations were examined by direct sequencing as previously described.^[22]^

### 2.4. Treatment

The patients in the cohort underwent surgery according to the standard protocol of our department. The minimum surgical extent for each patient was thyroid lobectomy and central lymph node dissection. If a thyroid nodule is also identified in the contralateral lobe, the surgeon, based on preoperative assessment and patient consultation, might perform a partial or subtotal lobectomy of the contralateral gland, or forgo treatment of the contralateral lobe. All excised suspicious carcinoma lesions were assessed by frozen section analysis. If the frozen section results indicated malignancy in the contralateral lobe nodes, contralateral lobectomy was continued.

### 2.5. Histopathologic examination

The pathologist identified the target tumor lesion’s location on the isolated specimen, based on the size and position of the suspicious nodule in the preoperative ultrasound report, made a pathologic diagnosis of the lesion, and thoroughly examined for the presence of additional cancerous lesions. Ipsilateral occult carcinoma was defined as tumor lesions other than the target tumor lesions discovered through postoperative pathology of the ipsilateral lobe. Contralateral occult carcinoma referred to a tumor lesion situated in the contralateral lobe, as identified by postoperative pathology. For lymph node dissection samples, the number of detected and metastatic lymph nodes was documented. In this study, the presence of occult lesions was defined as ipsilateral occult carcinoma, contralateral occult carcinoma, or lymph node metastases.

### 2.6. Statistical analysis

Continuous variables are presented as mean ± standard deviation (SD). Categorical variables were presented as percentages and compared using the chi-squared or Fisher’s exact tests, as suitable. Variables with *P* < .05 in the univariate analysis were incorporated into the logistic regression model employing the enter selection method. Results were expressed as odds ratios (OR) with 95% confidence intervals (CI) and *P* values. Statistical analyses were conducted using SPSS software (version 23.0, IBM, Armonk, New York, USA), and a *P* value of < .05 was statistically significant.

## 3. Result

This study included 413 patients. Two patients had Category VI FNA cytology with final postoperative pathology, one case of subacute thyroiditis, and one case of HT. Thirteen patients with Category V FNA cytology had a final pathology of nodular thyroid or subacute thyroiditis. These 15 patients were excluded from the follow-up analysis. An evaluation of the remaining 398 cases was conducted. The baseline clinicopathological characteristics of the patients are summarized in Table 1. Regarding FNA diagnostic outcomes, 75 patients were categorized as Category V, and 323 patients were Category VI. All patients were diagnosed with PTC on postoperative pathology, with 17 being follicular variant PTC and the remainder being classic PTC. Unilateral thyroid lobectomy and central lymph node dissection were performed in 143 patients, bilateral thyroidectomy and central lymph node dissection in 39, and partial or subtotal lobectomy of the contralateral lobe in 216. Three patients underwent unilateral lateral lymph node dissection due to intraoperative detection of multiple suspicious lymph nodes in the central compartment, with 3/37, 5/14, and 4/11 lateral lymph node metastases, respectively. The remaining 395 patients underwent central neck lymph node dissection alone.

**Table 1:** Clinicopathological characteristics of 398 PTMC patients.

Variables	N = 398 (%)
Sex	
Female	301 (75.6)
Male	97 (24.4)
Age (Y), Mean ± SD (range)	47.6 ± 11.7 (20–79)
<55	294 (73.9)
≥55	104 (26.1)
Tumor location	
Upper/Middle	302 (75.9)
Lower	96 (24.1)
Tumor sizes measured by preoperative US (mm), Mean ± SD (range)	6.3 ± 1.9 (2.4–10.0)
≤5	111 (27.9)
>5	287 (72.1)
Multiple nodules	
Absence	188 (47.2)
Presence	210 (52.8)
Irregular margin	
Absence	102 (25.6)
Presence	296 (74.4)
Taller than wide shape	
Absence	169 (42.5)
Presence	229 (57.5)
Multiple microcalcifications	
Absence	349 (87.7)
Presence	49 (12.3)
Adjacent to the capsule	
Absence	333 (83.7)
Presence	65 (16.3)
HT	
Absence	321 (80.7)
Presence	77 (19.3)
Tumor sizes measured by postoperative pathology (mm), Mean ± SD (range)	5.0 ± 2.4 (1–15)
≤5	263 (66.1)
> 5	135 (33.9)
Histological type	
Classical PTC	381 (95.7)
Follicular variant PTC	17 (4.3)
Ipsilateral occult carcinoma	
Absence	368 (92.5)
Presence	30 (7.5)
Contralateral occult carcinoma*	
Absence	227 (89.0)
Presence	28 (11.0)
CLNM	
Absence	304 (76.4)
Presence	94 (23.6)
No. of metastatic LNs in the central compartment, Mean ± SD (range)	0.5 ± 1.1 (0–9)
No. of removed LNs in the central compartment, Mean ± SD (range)	4.6 ± 3.4 (1–23)
BRAF V600E mutation	
Absence	77 (19.3)
Presence	321 (80.7)
TERT promoter mutation	
Absence	398 (100.0)
Presence	0 (0)

Categorical variables are presented as numbers (%, percentage).

CLNM = central lymph node metastasis, HT = Hashimoto’s thyroiditis, LNs = lymph nodes, PTMC = papillary thyroid microcarcinoma, US = ultrasonography.

* Contralateral occult carcinoma was only counted in 255 patients who had bilateral thyroid surgery.

**Figure 1.**
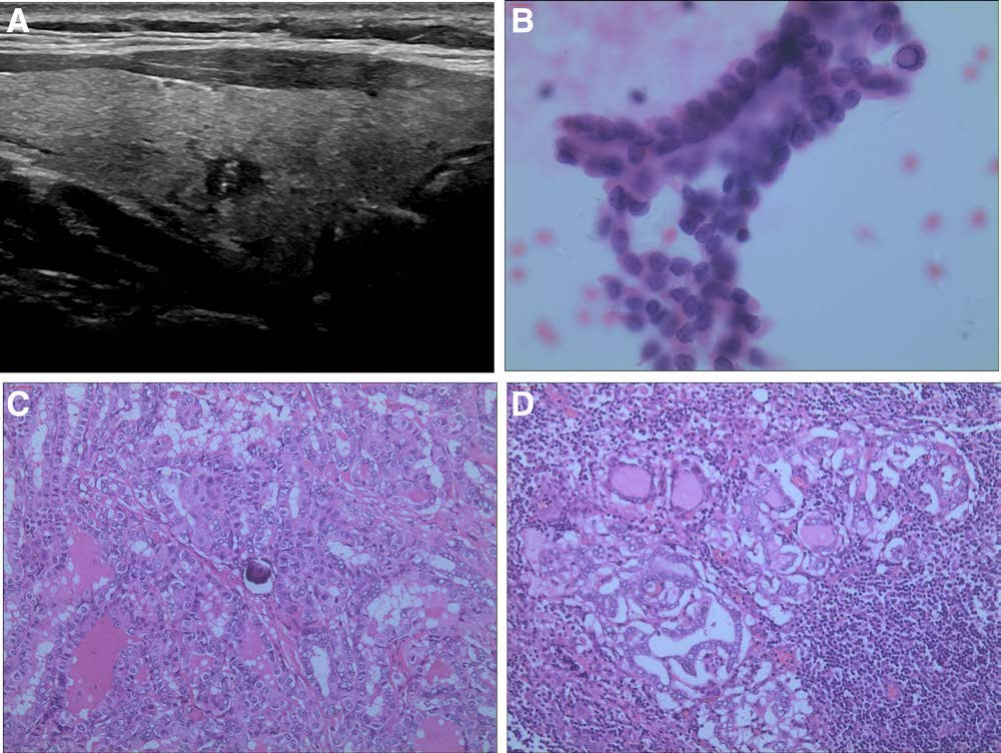
The ultrasound features, preoperative FNA cytology, and postoperative pathology of a 30-year-old male patient eligible for ablation. Preoperative ultrasound revealed a solitary 6-mm hypoechoic solid nodule with irregular margins and multiple punctate echogenic foci in the lower middle part of the right lobe, with no evidence of lymph node metastasis (A). FNA cytology of the nodule suggested a diagnosis of papillary carcinoma (Bethesda VI) (B, H&E staining, ×400). The patient underwent thyroid lobectomy and central lymph node dissection. Postoperative pathology confirmed a solitary papillary carcinoma in the right lobe with a maximum diameter of 4 mm (C, H&E staining, ×100). Two out of the 3 central lymph nodes that were dissected showed metastasis of papillary carcinoma (D, H&E staining, ×100). FNA = fine needle aspiration.

Among all the patients, 34.7% (138/398) presented with occult lesions other than the target tumor lesions. Among them, 7.5% (30/398) had ipsilateral occult carcinoma, averaging 1.1 ± 0.3 (range, 1–2) lesions per patient and a mean lesion size of 2.3 ± 1.6 mm (range, 1–8 mm). A total of 23.6% (94/398) patients had CLNM, with an average of 2.0 ± 1.4 (range, 1–9) metastases per patient. Among the 255 patients whose contralateral nodules were pathologically examined, 11.0% (28/255) had contralateral occult carcinoma, averaging 1.4 ± 0.7 (range, 1–4) lesions per patient and a mean lesion size of 3.1 ± 1.9 mm (range, 1–9 mm). Figure 1 shows preoperative ultrasonography, FNA cytology, and postoperative pathology in a representative case.

Molecular testing revealed that 80.7% of the patients exhibited the BRAF V600E mutation, with no detected TERT promoter mutations. According to the American Thyroid Association thyroid recurrence risk stratification, 6 cases were classified as intermediate-risk for postoperative recurrence, while the remaining patients were deemed low-risk, and no high-risk patients were identified.

Univariate analysis indicated that male gender, preoperative tumor size, tumor location in the lower pole, presence of multiple microcalcifications were risk factors for CLNM (*P* < .05). Multivariate analysis incorporating these factors demonstrated that male gender (OR: 2.188, 95% CI: 1.297–3.692, *P* = .003), preoperative tumor size (OR: 2.069, 95% CI: 1.113–3.845, *P* = .022), tumor location in the lower pole (OR: 1.893, 95% CI: 1.111–3.224, *P* = .019), and presence of multiple microcalcifications (OR: 2.810, 95% CI: 1.478–5.340, *P* = .002) were independent predictors of CLNM (Table 2).

**Table 2:** Associations between clinicopathological characteristics and CLNM in 398 PTMC patients.

Variables	CLNM (−)	CLNM (+)	*P* value	Multivariate analysis	
	N = 304 (76.4)	N = 94 (23.6)			
				OR (95% CI)	*P* value
Sex					
Female	241 (79.3)	60 (63.8)	**.002**	1 (reference)	**.003**
Male	63 (20.7)	34 (36.2)		2.188 (1.297–3.692)	
Age (Y)					
<55	222 (73.0)	72 (76.6)	.491		
≥55	82 (27)	22 (23.4)			
Tumor location					
Upper/Middle	239 (78.6)	63 (67.0)	**.022**	1 (reference)	**.019**
Lower	65 (21.4)	31 (33.0)		1.893 (1.111–3.224)	
Tumor sizes measured by preoperative US (mm)					
≤5	96 (31.6)	15 (16.0)	**.003**	1 (reference)	**.022**
>5	208 (68.4)	79 (84.0)		2.069 (1.113–3.845)	
Multiple nodules					
Absence	138 (45.4)	50 (53.2)	.186		
Presence	166 (54.6)	44 (46.8)			
Irregular margin					
Absence	76 (25.0)	26 (27.7)	.606		
Presence	228 (75.0)	68 (72.3)			
Taller than wide shape					
Absence	121 (39.8)	48 (51.1)	.054		
Presence	183 (60.2)	46 (48.9)			
Multiple microcalcifications					
Absence	277 (91.1)	72 (76.6)	**.000**	1 (reference)	**.002**
Presence	27 (8.9)	22 (23.4)		2.810 (1.478–5.340)	
Adjacent to the capsule					
Absence	254 (83.6)	79 (84.0)	.911		
Presence	50 (16.4)	15 (16.0)			
HT					
Absence	244 (80.3)	77 (81.9)	.723		
Presence	60 (19.7)	17 (18.1)			
BRAF V600E mutation					
Absence	55 (18.1)	22 (23.4)	.254		
Presence	249 (81.9)	72 (76.6)			

Categorical variables are presented as numbers (%, percentage). Values in bold indicate statistically significant differences at the *P* < 0.05 level.

CLNM = central lymph node metastasis, HT = Hashimoto’s thyroiditis, PTMC = papillary thyroid microcarcinoma, US = ultrasonography.

Univariate analysis demonstrated that multiple microcalcifications and HT were risk factors for ipsilateral occult carcinoma (*P* < .05). Multivariate analysis of these 2 factors revealed that multiple microcalcifications (OR: 4.929, 95% CI: 2.081–11.673, *P* = .000) and HT (OR: 3.629, 95% CI: 1.611–8.174, *P* = .002) were independent predictors of ipsilateral occult carcinoma (Table S1, Supplemental Digital Content, http://links.lww.com/MD/J718).

The association between clinicopathological characteristics and contralateral occult carcinoma was investigated in 255 patients with PTMC whose contralateral thyroid nodules were also examined pathologically. Univariate analysis indicated that multiple microcalcifications and HT were risk factors for contralateral occult carcinoma (*P* < .05). Multivariate analysis of these 2 factors demonstrated that multiple microcalcifications (OR: 7.085, 95% CI: 2.693–18.640, *P* = .000) and HT (OR: 4.542, 95% CI: 1.847–11.172, *P* = .001) were independent predictive factors for contralateral occult carcinoma (Table S2, Supplemental Digital Content, http://links.lww.com/MD/J719).

Upon combining patients with ipsilateral occult carcinoma, contralateral occult carcinoma, and lymph node metastases, collectively denoted as patients with “occult lesions,” a re-assessment indicated that male gender (OR: 2.526, 95% CI: 1.521–4.195, *P* = .000), tumor location in the lower pole (OR: 1.969, 95% CI: 1.186–3.267, *P* = .009), multiple microcalcifications (OR: 5.620, 95% CI: 2.837–11.134, *P* = .000), and HT (OR: 2.245, 95% CI: 1.292–3.899, *P* = .004) were independent predictors of occult lesions (Table 3).

**Table 3:** Associations between clinicopathological characteristics and occult lesions in 398 PTMC patients.

Variables	Occult lesions (−)	Occult lesions (+)	*P* value	Multivariate analysis	
	N = 260 (65.3)	N = 138 (34.7)		OR (95% CI)	*P* value
Sex					
Female	211 (80.8)	91 (65.9)	**.001**	1 (reference)	**.000**
Male	50 (19.2)	47 (34.1)		2.526 (1.521–4.195)	
Age (Y)					
<55	195 (75.0)	99 (71.7)	.481		
≥55	65 (25.0)	39 (28.3)			
Tumor location					
Upper/middle	209 (80.4)	93 (67.4)	**.004**	1 (reference)	**.009**
Lower	51 (19.6)	45 (32.6)		1.969 (1.186–3.267)	
Tumor sizes measured by preoperative US (mm)					
≤5	84 (32.3)	27 (19.6)	**.007**	1 (reference)	.090
>5	176 (67.7)	111 (80.4)		1.577 (0.932–2.669)	
Multiple nodules					
Absence	125 (48.1)	63 (45.7)	.645		
Presence	135 (51.9)	75 (54.3)			
Irregular margin					
Absence	65 (25.0)	37 (26.8)	.694		
Presence	195 (75.0)	101 (73.2)			
Taller than wide shape					
Absence	104 (40.0)	65 (47.1)	.173		
Presence	156 (60.0)	73 (52.9)			
Multiple microcalcifications					
Absence	245 (94.2)	104 (75.4)	**.000**	1 (reference)	**.000**
Presence	15 (5.8)	34 (24.6)		5.620 (2.837–11.134)	
Adjacent to the capsule					
Absence	215 (82.7)	118 (85.5)	.470		
Presence	45 (17.3)	20 (14.5)			
HT					
Absence	219 (84.2)	102 (73.9)	**.013**	1 (reference)	**.004**
Presence	41 (15.8)	36 (26.1)		2.245 (1.292–3.899)	
BRAF V600E mutation					
Absence	50 (19.2)	27 (19.6)	.936		
Presence	210 (80.8)	111 (80.4)			

Categorical variables are presented as numbers (%, percentage). Values in bold indicate statistically significant differences at the *P* < 0.05 level.

HT = Hashimoto’s thyroiditis, PTMC = papillary thyroid microcarcinoma, US = ultrasonography.

**Figure 2.**
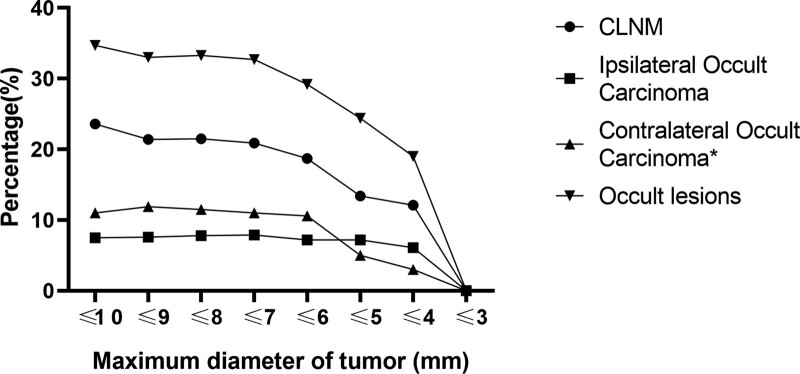
The likelihood of occult lesions in patients with varying tumor sizes. *Contralateral occult carcinoma was only counted in 255 patients who had bilateral thyroid surgery.

Based on the maximum tumor diameter displayed in preoperative ultrasound, the likelihood of occult lesions in patients with varying tumor sizes was analyzed using ≤ 10, ≤9, ≤8, ≤7, ≤6, ≤5, ≤4, and ≤ 3 mm as intercept points; as demonstrated in Figure 2, when the maximum tumor diameter was ≤ 6 mm, the probability of occult lesions progressively diminished; when the maximum tumor diameter was ≤ 3 mm, the probability of occult malignancy was 0.

## 4. Discussion

In our investigation, we discovered that among low-risk PTMC patients with tumor characteristics suitable for thermal ablation, 34.7% may have occult lesions other than the target tumor lesions, encompassing ipsilateral or contralateral occult carcinoma or lymph node metastases, if only treated locally. Male gender, tumor location in the lower pole, multiple microcalcifications, and HT were independent risk factors for the presence of occult lesions in patients.

Lymph node metastasis in the central compartment of cN0 PTMC is relatively common. Previous studies indicated a 25.9% to 49.2% likelihood of metastases and associations with male gender, multifocality, tumor size > 5 mm, and extrathyroidal extension.^[23]^ In our cohort, the preoperatively enrolled patients were considered to have a single lesion without thyroid capsular invasion or extrathyroidal extension. The incidence of CLNM in our population was 23.6%, which was lower than the previously reported average. In line with prior studies, our research identified male gender and tumor size > 5 mm as independent risk factors for CLNM and identified tumor location in the lower pole and multiple microcalcifications as independent risk factors for CLNM. A retrospective analysis of 1066 PTMC cases by Zhang et al revealed that tumors situated in the lower third of the thyroid had a 14-fold higher risk of CLNM than the upper third and a 4-fold higher risk of CLNM than the middle third.^[24]^ This may be attributed to the location of lymphatic drainage areas in various thyroid gland regions.^[25]^ On ultrasound images, microcalcifications are defined as hyper-echoic punctate foci ≤ 1 mm, while on pathology, they reflect psammoma bodies, which are significantly associated with lymph node metastasis.^[26]^ In this study, the likelihood of CLNM was notably greater in patients exhibiting multiple microcalcifications than in those without them. Similarly, Zhang et al discovered a significant association between clustered calcification and CLNM metastasis in PTC.^[27]^

PTC frequently manifest as multifocal or bilateral lesions. Among patients undergoing total thyroidectomy for preoperatively detected unilateral PTMC, 15.5% to 40.4% exhibit contralateral occult carcinoma.^[28–31]^ Even with preoperative solitary PTMC consideration, 3.7% of patients develop ipsilateral occult carcinoma, and 15.5% to 22.3% contralateral occult carcinoma.^[32,33]^ In this investigation, 11.0% (28/255) of patients had contralateral occult carcinoma. Since most enrolled patients with contralateral nodules underwent only partial or subtotal excision, and those with preoperative suspicion of unilateral multiple foci were excluded, the probability of contralateral occult carcinoma was relatively low. Nevertheless, preoperative HT diagnosis and multiple microcalcifications in the primary tumor have been identified as high-risk factors for ipsilateral and contralateral occult carcinoma development. The association between HT and PTC is controversial, but many studies have reported an increased multifocal PTC incidence and a higher tendency for occult cancer development in HT patients.^[31,34,35]^ Thus, total thyroidectomy may be a safer and more comprehensive treatment for HT patients to avoid undetected occult foci. Similar to CLNM, multiple microcalcifications at the primary site independently increased the risk of occult carcinoma. This implies that a greater number and distribution of punctate echogenic foci could be linked to PTC’s aggressive biological behavior,^[36]^ warranting further research.

pretreatment prognostic evaluation of PTMC is crucial for treatment selection. The molecular testing of FNA samples is promising. The BRAF V600E mutation has been extensively studied as a prognostic biomarker for PTMC, but its role in risk stratification remains debatable. A meta-analysis of 33 studies revealed that among 8838 patients, 5043 (57.1%) had BRAF V600E mutations, and these patients exhibited a higher risk of multifocality, extrathyroidal extension, lymph node metastasis, and disease recurrence.^[37]^ However, the study included diverse ethnic groups, resulting in genetic background variations. Regarding BRAF V600E mutation rates, East Asian populations, such as China and Korea, had significantly higher rates, which is consistent with other studies.^[38,39]^ In a Chinese population-based study with similar findings, BRAF V600E mutations were detected in 249 PTMC patients (83.3%, 249/299), and univariate analysis indicated no significant association between CLNM, lateral lymph node metastasis, multifocality, extrathyroidal extension, recurrence, and BRAF V600E mutations.^[40]^ Additional research is required to determine whether isolated BRAF V600E mutations in cytology samples can serve as prognostic markers to guide low-risk PTMC treatments across different ethnic populations.

TERT promoter mutations have been recognized as potent prognosticators of adverse outcomes in thyroid neoplasms. Patients with PTC exhibiting concurrent BRAF V600E and TERT promoter mutations tend to have a poorer prognosis.^[41]^ Nevertheless, the prevalence of TERT promoter mutations is lower in PTMC than in larger PTC. Song et al conducted a retrospective analysis of the TERT promoter mutation status of 3435 PTC patients, stratified into subgroups based on tumor size, revealing that the mutation frequency in the TERT promoter was reduced in all 3 subgroups with tumors ≤ 1 cm and increased significantly with tumor size.^[42]^ Additional studies revealed that the TERT promoter mutation frequency was considerably lower in PTMC (0–4.7%) than in PTC.^[43–45]^ In the present study, no TERT promoter mutations were detected. Yabuta et al^[46]^ also identified no TERT promoter mutations in specimens from low-risk PTMC patients undergoing conversion surgery following active surveillance, suggesting the limited utility of TERT mutation detection in low-risk PTMC cases.

Regarding postoperative pathological evaluation, most low-risk PTMC patients subjected to thermal ablation experience safety and may even attain surgical curative efficacy, provided that thermal ablation indications are rigorously controlled. In our study cohort, 98.5% of patients were classified as American Thyroid Association low-risk, only 6 patients were categorized as intermediate-risk postoperatively, and no high-risk patients were identified; only 3 patients required lateral lymph node dissection in the neck. Although 34.7% of patients exhibited occult lesions, the average diameter of these occult lesions was relatively small, with an average of 2 lymph node metastases. As the majority of PTMC cases exhibit slow progression, these residual lesions may elevate local recurrence rates later; however, their impact on patients’ quality of life or disease survival necessitates long-term follow-up.^[47]^ Consequently, before conducting thermal ablation, patients must be thoroughly informed of the benefits and limitations of this novel treatment modality and should have a comprehensive understanding of their anticipated disease treatment outcomes.

This study has certain limitations. First, since the contralateral thyroid gland was only partially or subtotally resected in most patients, the actual incidence of contralateral occult carcinoma may be higher than the reported probability. Second, this investigation was a simulation study; in actual ablated patients, the thermal ablation range will be expanded, with the surrounding thermal radiation effect on the tumor potentially inactivating some satellite foci immediately adjacent to the ablated lesion, thereby reducing the actual ipsilateral tumor residual rate compared to this simulated study. Third, this study solely encompasses postoperative pathological analysis; the extent to which these subclinical lesions will influence future disease recurrence and patient survival in real-world practice necessitates the implementation of clinical thermal ablation and long-term follow-up.

## 5. Conclusion

In low-risk PTMC patients exhibiting tumor characteristics that are amenable to thermal ablation, over one-third may present with occult lesions. Prior to initiating thermal ablation therapy, thorough patient communication regarding the advantages and disadvantages of this treatment modality is imperative, along with a clear understanding of the patient’s therapeutic expectations. It is essential to inform the patient that the treatment objective is not tumor eradication, but rather disease progression prevention and surgical intervention avoidance. Meticulous evaluation of the presence of additional lesions is necessary before performing thermal ablation, particularly in patients exhibiting high-risk factors for occult lesions. Post-thermal ablation, regular follow-up is mandated to vigilantly monitor any increase in occult lesions.

## Author contributions

**Data curation:** Langping Jin, Kaijun Zhu, Changliang Xu, Jiaying Lu.

**Formal analysis:** Changliang Xu, Jiaying Lu.

**Project administration:** Liming Huang.

**Supervision:** Liming Huang.

**Writing – original draft:** Langping Jin, Kaijun Zhu.

**Writing – review & editing:** Liming Huang.

## Supplementary Material




