# Supplementary material for: Incidence and risk factors for occult lesions in low-risk papillary thyroid microcarcinoma patients with tumor characteristics appropriate for thermal ablation: A retrospective study
Source: Medicine (Baltimore). 2023 Sep 22;102(38):e34938. doi: 10.1097/MD.0000000000034938 (PMC10519479; doi:10.1097/MD.0000000000034938)
Supplement: Supplementary file 1 [file medi-102-e34938-s001.docx]

Supplementary Table 1. Associations between clinicopathological characteristics and ipsilateral occult carcinoma in 398 PTMC patients.

| Variables | Ipsilateral occult (-) | Ipsilateral occult (+) | P-value | Multivariate analysis | |
| --- | --- | --- | --- | --- | --- |
|  | N=368 (92.5) | N=30 (7.5) |  | OR (95% CI) | P-value |
| **Sex** |  |  |  |  |  |
| Female | 279 (75.8) | 22 (73.3) | .761 |  |  |
| Male | 89 (24.2) | 8 (26.7) |  |  |  |
| **Age (Y)** |  |  |  |  |  |
| <55 | 272 (73.9) | 22 (73.3) | .945 |  |  |
| ≥55 | 96 (26.1) | 8 (26.7) |  |  |  |
| **Tumor location** |  |  |  |  |  |
| Upper/Middle | 281 (76.4) | 21 (70.0) | .434 |  |  |
| Lower | 87 (23.6) | 9 (30.0) |  |  |  |
| **Tumor sizes measured by preoperative US (mm)** |  |  |  |  |  |
| ≤5 | 101 (27.4) | 10 (33.3) | .489 |  |  |
| >5 | 267 (72.6) | 20 (66.7) |  |  |  |
| **Multiple nodules** |  |  |  |  |  |
| Absence | 178 (48.4) | 10 (33.3) | .113 |  |  |
| Presence | 190 (51.6) | 20 (66.7) |  |  |  |
| **Irregular margin** |  |  |  |  |  |
| Absence | 93 (25.3) | 9 (30.0) | .568 |  |  |
| Presence | 275 (74.7) | 21 (70.0) |  |  |  |
| **Taller than wide shape** |  |  |  |  |  |
| Absence | 156 (42.4) | 13 (43.3) | .920 |  |  |
| Presence | 212 (57.6) | 17 (56.7) |  |  |  |
| **Multiple microcalcifications** |  |  |  |  |  |
| Absence | 329 (89.4) | 20 (66.7) | **.001** | 1 (reference) | **.000** |
| Presence | 39 (10.6) | 10 (33.3) |  | 4.929 (2.081-11.673) |  |
| **Adjacent to the capsule** |  |  |  |  |  |
| Absence | 308 (83.7) | 25 (83.3) | 1.000 |  |  |
| Presence | 60 (16.3) | 5 (16.7) |  |  |  |
| **HT** |  |  |  |  |  |
| Absence | 305 (82.9) | 19 (63.3) | **.008** | 1 (reference) | **.002** |
| Presence | 63 (17.1) | 11 (36.7) |  | 3.629 (1.611-8.174) |  |
| **BRAF V600E mutation** |  |  |  |  |  |
| Absence | 74 (20.1) | 3 (10.0) | .178 |  |  |
| Presence | 294 (79.9) | 27 (90.0) |  |  |  |

*Categorical variables are presented as numbers (%, percentage).*

*PTMC, papillary thyroid microcarcinoma; US, ultrasonography; HT, Hashimoto's thyroiditis.*
