# Supplementary material for: Incidence and risk factors for occult lesions in low-risk papillary thyroid microcarcinoma patients with tumor characteristics appropriate for thermal ablation: A retrospective study
Source: Medicine (Baltimore). 2023 Sep 22;102(38):e34938. doi: 10.1097/MD.0000000000034938 (PMC10519479; doi:10.1097/MD.0000000000034938)
Supplement: Supplementary file 2 [file medi-102-e34938-s002.docx]

Supplementary Table 2. Associations between clinicopathological characteristics and contralateral occult carcinoma in 255 PTMC patients.

| Variables | Contralateral occult (-) | Contralateral occult (+) | P value | Multivariate analysis | |
| --- | --- | --- | --- | --- | --- |
|  |  |  |  | OR (95% CI) | P-value |
|  | N=227 (89.0) | N=28 (11.0) |  |  |  |
| **Sex** |  |  |  |  |  |
| Female | 173 (76.2) | 21 (75.0) | .887 |  |  |
| Male | 54 (23.8) | 7 (25.0) |  |  |  |
| **Age (Y)** |  |  |  |  |  |
| <55 | 155(68.3) | 17 (60.7) | .420 |  |  |
| ≥55 | 72 (31.7) | 11 (39.3) |  |  |  |
| **Tumor location** |  |  |  |  |  |
| Upper/Middle | 167 (73.6) | 20 (71.4) | .809 |  |  |
| Lower | 60 (26.4) | 8 (28.6) |  |  |  |
| **Tumor sizes measured by preoperative US (mm)** |  |  |  |  |  |
| ≤5 | 72 (31.7) | 4 (14.3) | .057 |  |  |
| >5 | 155 (68.3) | 24 (85.7) |  |  |  |
| **Irregular margin** |  |  |  |  |  |
| Absence | 57 (25.1) | 8(28.6) | .692 |  |  |
| Presence | 170 (74.9) | 20 (71.4) |  |  |  |
| **Taller than wide shape** |  |  |  |  |  |
| Absence | 85(37.4) | 12 (42.9) | .578 |  |  |
| Presence | 142 (62.6) | 16(57.1) |  |  |  |
| **Multiple microcalcifications** |  |  |  |  |  |
| Absence | 205 (90.3) | 18 (64.3) | **.001** | 1 (reference) | **.000** |
| Presence | 22 (9.7) | 10 (35.7) |  | 7.085(2.693-18.640) |  |
| **Adjacent to the capsule** |  |  |  |  |  |
| Absence | 195 (85.9) | 26 (92.9) | .392 |  |  |
| Presence | 32 (14.1) | 2 (7.1) |  |  |  |
| **HT** |  |  |  |  |  |
| Absence | 185 (81.5) | 16 (57.1) | **.003** | 1 (reference) | **.001** |
| Presence | 42 (18.5) | 12 (42.9) |  | 4.542 (1.847-11.172) |  |
| **BRAF V600E mutation** |  |  |  |  |  |
| Absence | 47 (20.7) | 5 (17.9) | .724 |  |  |
| Presence | 180 (79.3) | 23 (82.1) |  |  |  |

*Categorical variables are presented as numbers (%, percentage).*

*PTMC, papillary thyroid microcarcinoma; US, ultrasonography; HT, Hashimoto's thyroiditis.*
